# Evaluation of High-Resolution Precipitation Estimates from Satellites during July 2012 Beijing Flood Event Using Dense Rain Gauge Observations

**DOI:** 10.1371/journal.pone.0089681

**Published:** 2014-04-01

**Authors:** Sheng Chen, Huijuan Liu, Yalei You, Esther Mullens, Junjun Hu, Ye Yuan, Mengyu Huang, Li He, Yongming Luo, Xingji Zeng, Guoqiang Tang, Yang Hong

**Affiliations:** 1 School of Civil Engineering and Environmental Science, University of Oklahoma, Norman, Oklahoma, United States of America; 2 Advanced Radar Research Center, National Weather Center, Norman, Oklahoma, United States of America; 3 Anhui Weather Modification Office, Hefei, China; 4 Departments of Earth, Ocean, and Atmospheric Science, Florida State University, Tallahassee, Florida, United States of America; 5 Cooperative Institutes for Mesoscale Meteorological Studies, University of Oklahoma, Norman, Oklahoma, United States of America; 6 School of Meteorology, University of Oklahoma, Norman, Oklahoma, United States of America; 7 School of Computer Science, University of Oklahoma, Norman, Oklahoma, United States of America; 8 Beijing Weather Modification Office, Beijing, China; 9 Remote Sensing Application Test Base of National Satellite Center, Gaugnxi, China; 10 Disaster Mitigation Institute of the Guangxi Meteorological Bureau, Nanning, Gaugnxi, China; 11 Meteorological Information Center of the Guangxi Meteorological Bureau, Nanning, Gaugnxi, China; 12 Department of Hydraulic Engineering, Tsinghua University, Beijing, China; NASA Jet Propulsion Laboratory, United States of America

## Abstract

Satellite-based precipitation estimates products, CMORPH and PERSIANN-CCS, were evaluated with a dense rain gauge network over Beijing and adjacent regions for an extremely heavy precipitation event on July 21 2012. CMORPH and PEERSIANN-CSS misplaced the region of greatest rainfall accumulation, and failed to capture the spatial pattern of precipitation, evidenced by a low spatial correlation coefficient (CC). CMORPH overestimated the daily accumulated rainfall by 22.84% while PERSIANN-CCS underestimated by 72.75%. In the rainfall center, both CMORPH and PERSIANN-CCS failed to capture the temporal variation of the rainfall, and underestimated rainfall amounts by 43.43% and 87.26%, respectively. Based on our results, caution should be exercised when using CMORPH and PERSIANN-CCS as input for monitoring and forecasting floods in Beijing urban areas, and the potential for landslides in the mountainous zones west and north of Beijing.

## Introduction

On July 21, 2012, Beijing experienced one of the heaviest rain events in the past 60 years. The heavy rainfall triggered flash flooding and landslides, which killed 79 people and caused US $2 billion in direct economic losses, destroying at least 8,200 homes in the city and affecting more than 1.6 million people [1], [2]. A Study by [3] indicated that this extreme event was the result of a frontal system and mid-troposphere disturbance. Sang et al. [4] pointed out that topographic effects and natural climate variability, coupled with a changing climate system, may have contributed to the severity of this precipitation event. Additionally, low standards for mountain torrents monitoring and control for medium and small rivers in the effected regions contributed to the high-impact flooding. The inhomogeneous precipitation, both spatially and temporally, as well as excessive runoff due to increasing impervious surface areas, contributed to severe waterlogging in the urban area in Beijing.

Satellite-based quantitative precipitation estimates (QPE) products are widely applied to hydrologic modeling, hazards monitoring and climate research due to their global coverage and spatial continuity [5], [6], [7], [8], [9], [10]. Thermal Infrared (IR) and passive microwave (PMW) sensors are the most widely used instruments to quantitatively estimate rainfall on the ground. The physical bases of rainfall estimation from both IR and PMW sensors imagery are well explored and explained by many previous works [11], [12], [13]. Rainfall estimation from IR sensors depends on the assumption that the surface rainfall can be inferred from analyzing the cloud top characteristics, i.e., the cloud top temperature. That is, where the clouds which can reach highest altitude, and therefore have the lowest cloud top temperature are the most probably to precipitate. On the other hand, passive microwave measurements provide more physically direct link between hydrometeors in the atmosphere and radiances observed from a satellite. While being different in details, the PMW algorithms estimate rainfall rates fundamentally under the same principle: translating the scattering signature caused by ice water and/or emission signature caused by liquid water into a surface rainfall rate. Although the PMW sensors have clear advantages for rainfall estimation since they provide more information from the hydrometers themselves in the air, the IR sensors usually have much higher spatio-temporal resolution. Therefore, many recent rainfall algorithms utilize brightness temperatures from both PMW and IR sensors to achieve better rainfall estimation accuracy. Algorithms which combine both the advantages of PMW and IR were developed to estimate the rainfall [14], [15], [16], [17], [18], for example, the IR-dominated Precipitation Estimation from Remotely Sensed Information using Artificial Neural Networks(ANN) [16] and later the PERSIANN-Cloud Classification System (PERSIANN-CSS) [18], the National Oceanic and Atmospheric Administration's(NOAA) Climate Prediction Center(CPC) morphing technique(CMORPH) [14]. Studies by Dinku et al. [19] and [20] shows that the CMORPH and PERSIANN-CCS underestimated the rainfall by more than 30% in extreme precipitation events.

This study aimed to evaluate the performance of algorithms PERSIANN-CCS and CMORPH with a relatively dense rain gauge network over Beijing and the surrounding regions for the July 21, 2012 extreme precipitation event. The paper is organized as follows. Section 2 describes the study region and the evaluated rainfall products. Section 3 provides an analysis of spatial characteristics and error quantification for the rainfall from 00:00AM on July 21 to 00:00PM on July 22 2012. The total rainfall accumulation, the temporal variation of mean hourly rainfall, and rainfall contingency as a function of rainfall intensity are described in this section. A summary of analysis results and conclusions are provided in Section 4.

## Study Region and Data

### 2.1 Study region

**Figure 1:**
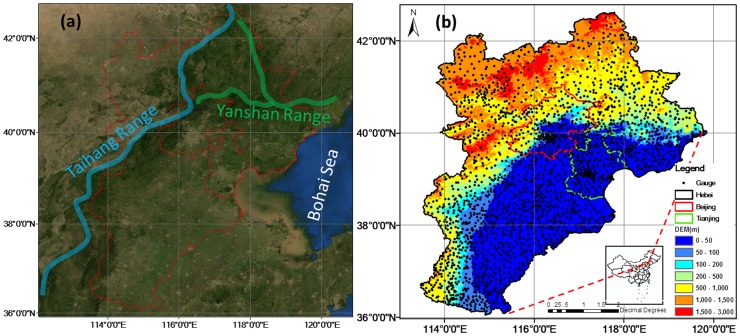
Study area (a) outlined in red lines and Taihang and Yanshan Ranges distribution on the remote sensing map from the ArcGIS Map Service. (b) Gauge distribution and topographic features in Beijing, Tianjin and Hebei province.

The study region, including Hebei province, Beijing and Tianjin, spans from 36°′N to 42.5°N in latitude and from 113.5°08′E to 120°E in longitude (Fig. 1). The east of study region terminates at the Bohai Sea, the western at the Taihang mountain ridge, and the northern at the Yanshan mountain ridge.

### 2.2 Data

Dense Automatic Weather Stations (AWS) gauge network observations from 2401 stations (Figure 1b) are used as the ‘ground truth’. The gauges are part of an enhanced dense gauge network over China, providing hourly real-time weather observations for authorized natural hazard monitoring departments and weather service sectors. An analysis by Ren [21] shows that the average rainfall error of gauge observations is 6.52% [32], which is much smaller than satellite-based precipitation estimates. The satellite-based quantitative precipitation estimates (QPE) products include hourly PERSIANN-CCS with a spatial resolution 0.04° and 3-hourly CMORPH with a spatial resolution 0.25°.

PERSIAN-CCS is an automated system for precipitation estimation from remotely sensed information using the artificial neural network, which extracts local and regional cloud features from infrared geostationary satellite imagery to estimate rainfall distribution. The system retrieves different rainfall rates at a given brightness temperature (*T_b_*) and detects variable rain/no-rain IR thresholds for different cloud types [18], [22]. In this study, PERSIANN-CCS is resampled from 0.04° to 0.25° in order to compare with CMPORH at the same scale.

CMORPH has a quasi-global coverage (60°S-60°N in latitude) and is of very high-resolution (0.07278°, approximately 8 km, in latitude/longitude, half-hourly) [14]. Motion vectors derived from the half-hourly geostationary satellite IR imagery are used to extrapolate the passive microwave precipitation estimates. This technique improves the estimation of multi-hour precipitation accumulation, better than the simple averaging of available microwave-based estimates and other merging results that incorporate microwave and infrared information in the estimation [14]. Only the most recent one-month half-hourly/0.07278° CMORPH product could be publically accessed, with data during the July 21 Beijing flood event not available when this work was initiated in October of 2012. Therefore, the 3 hourly/0.25° CMORPH product was used together with PERSIANN-CCS to compare with the gauge observations.

### 2.3 Statistics metrics

Statistic metrics include Relative bias (RB), root-mean-squared error (RMSE), correlation coefficient (CC), probability of detection (POD), false alarm ratio (FAR) and critical success index (CSI) are used in this study to evaluate the performance of all the rainfall algorithms. These metrics are defined as follows.
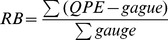
(1)

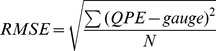
(2)

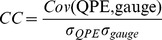
(3)where RB and CC are dimensionless, RMSE is in mm. In Eq. (3), “Cov ()” refers to the covariance, and *σ* indicates the standard deviation. RB, when multiplied by 100, denotes the degree of overestimation or underestimation in percentage. All the above statistics were computed for each pixel location within the study region.

Table 1 provides the contingency table to compute the number of hits (A), the false alarm (B), and miss rate (C). Statistics of POD, FAR and CSI were computed with the following equations [19]:
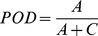
(4)

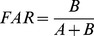
(5)

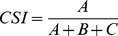
(6)


**Table 1:** Contingency table comparing gauge area-averages and satellite rainfall estimates.

	Gauge≥Threshold	Gauge<Threshold
Satellite≥Threshold	A	B
Satellite<Threshold	C	D

## Analysis of Rainfall Products

### 3.1 Accumulated rainfall

**Figure 2:**
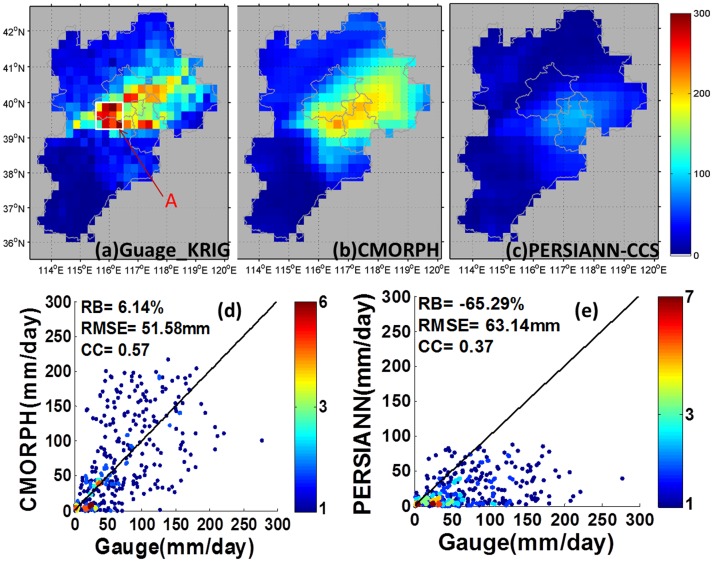
Gauge analysis (a) based on Kriging interpolation. (b) CMORPH accumulated precipitation (c) PERSIANN-CCS accumulated precipitation. (d) Scatter plots of gauge vs. CMORPH accumulated rainfall. (e) Scatter plot of gauge vs. PERSIANN-CCS accumulated rainfall. The red letter “A” in (a) indicates the rainfall center.

Rainfall estimates from all the products were accumulated from 00:00AM on 21 July 2012 to 00:00AM on 22 July 2012 to yield a two-day total rainfall as shown in Figure 2. The gauge daily observations were interpolated over the study domain based on Kriging interpolation skill, which is embedded in the Interactive Data Language (IDL, Version 8.2). The exponential model and default variogram model were used for the interpolation. The interpolated result (Figure 2(a)) indicates the maximum in accumulated rainfall extending from southwest of Beijing northeastward all the way to the adjacent Beijing and Hebei province, and eastward to center of Tianjin. CMORPH and PERSIANN-CCS misplaced this precipitation maximum. It is also worth noting that PERSIANN-CCS underestimated the rainfall by a wide margin, with maximum rainfall less than 200 mm.

**Figure 3:**
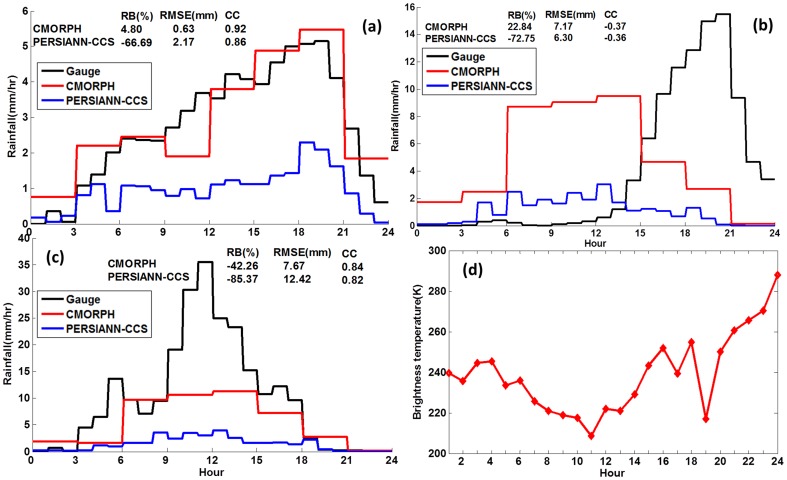
Time series rainfall over rainfall center of (a) large region HBT, (b) Beijing and (c) rainfall center. (d) Time series mean brightness temperature at rainfall center shown in Figure 1d in the scale.

For quantitative comparison, only the grids that cover two and more rain gauges were used for comparison. Grids containing less than two gauges were discarded. Such grid-based comparison technique has been applied in many previous studies [23], [24], [25], [26], [27]. A total of 345 grids were available for direct comparison with a 0.25°×0.25° spatial resolution for the large region including Hebei, Beijing and Tianjin, and 30 grids for Beijing. Figure 2d–e show that both CMORPH and PERSIANN-CCS have low CC with gauge-based observations, which indicates CMORPH and PERSIANN-CCS failed to capture the spatial rainfall pattern. The index RB implies CMORPH overestimated the rainfall by about 6.14% but PERSIANN-CCS significantly underestimated the rainfall by 65.29%. Figure 3d indicates the cloud top in the region of highest accumulated precipitation was above 220 k except for the 11^th^ hour. This suggests the IR-based QPE retrieve technique has inherent shortage that using IR brightness temperatures to estimate precipitation will yield a large error when the convective cloud has a higher brightness temperature than the commonly used temperature threshold [28], [29]. For CMORPH, the misplacement of the most intense rainfall might be due to: 1) Precipitation that is not observed, as it forms and dissipates over an area outside of passive microwave overpasses mosaics; or 2) Ice areas assigned zero rainfall estimates by the snow screening process in the CMORPH algorithm [14], [20]. This result is consistent with the findings in Hirpa et al. [30], Zhang [20] and Chen et al. [27] over complex terrain during heavy precipitation events.

### 3.2 Precipitation time-series

Figure 3 shows time series plots of hourly area-average accumulated rainfall for gauge and satellite products for the large domain including Hebei, Beijing and Tianjin (HBT), and Beijing and the rainfall center indicated by the red letter A in Figure 2a. In Figure 3a, the gauge observed rainfall over HBT generally increased from the first hour to the peak occurring at the 20^th^ hour and then decreased dramatically to less than 1 mm/h by the 24^th^ hour. CMORPH captured the peak of rainfall and generally replicated the temporal evolution observed by the gauge. PERSIANN-CCS produced a peak in rainfall one hour ahead of the gauge but substantially underestimated the amounts throughout the event. The statistics show that CMORPH had a high CC of 0.92 and low RB (4.80%), while PERSIANN-CCS had a slightly lower CC (0.86) but a highly negative RB (−66.69%). For the Beijing area (Figure 3b), CMORPH and particularly PERSIANN-CCS failed to capture the rainfall peak and temporal variation, evidenced by the negative CC. Furthermore, CMORPH generally overestimated the rainfall, while PERISANN-CCS underestimated by −72.75%. For the rainfall maximum in the rainfall center (Figure 3c), CMORPH and PERSIANN-CCS performed poorly in capturing the rainfall peak and temporal variation, underestimating total accumulation by 42.26% and 85.37%, respectively.

It was noted that in the region of highest rainfall accumulation the gauge-observed very heavy rainfall (>15 mm/h) began at the 9^th^ hour and lasted to the 15^th^ hour. This rainfall intensity exceeded the lower bound of the rainfall intensity-duration threshold (Intensity = 12.45 mm/h Duration = 0.42 h) for landslides proposed by Hong et al. [31]. Several mountain torrents, floods, and landslides took place in these areas (http://www.youtube.com/watch?v=X1gzzUipevg; http://video.lnd.com.cn/htm/2012-07/25/content_2422846.htm), and 38 people were killed in Fangshan County, located in the rainfall maximum. Compared with gauge observation, the most intense rainfall observed by both CMORPH and PERISANN-CCS did not exceed 12.45 mm/h. Therefore, the landslide and debris would not have triggered if CMORPH and PERISANN-CCS were used as input to the landslide model proposed by Hong et al. [31].

### 3.3 Probability distributions

**Figure 4:**
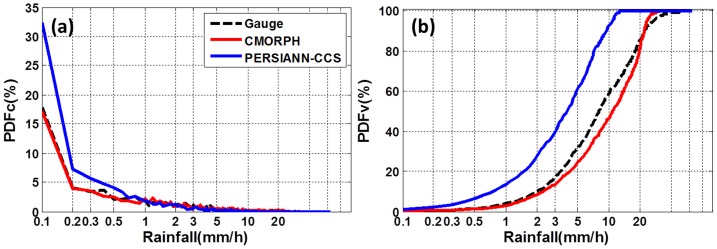
Occurrence probability distribution (a) of rain rate with interval of 0.1 mm/h, (b) volume cumulative distribution of rain rate with interval of 0.1 mm/h.

Information on the precipitation rate distribution, precipitation volume distribution, and the precipitation estimates' sensitivity as a function of precipitation rate may be revealed with Probability distribution functions (PDFs). PDFs offers insight into error dependence on precipitation rate, and the potential impact of this error on hydrological applications [32]. Figure 4 shows the hourly mean precipitation PDFs by occurrence (PDFc) and cumulative distribution of rainfall rate by volume (CDFv) for the gauge and satellite-based observations. Figure 4a shows that CMORPH had the best agreement with gauge observations in terms of hourly mean precipitation occurrence, while PERSIANN-CCS had a higher percentage of light rainfall rates (<1 mm/h), and low percentages for high rainfall rate (>5 mm/h). Figure 4b shows that more than 90% of the total rainfall accumulation derived from PERSIANN-CSS was contributed by rainfall rates less than 10 mm/h. The distributions for CMORPH and PERSIANN-CSS were similar for rainfall rates less than 22 mm/h; however, the gauge observations had more contribution from rainfall rates greater than 22 mm/h, especially relative to PERSIANN-CSS. This implies that CMORPH and PERSIANN-CCS could not resolve the intense rainfall rates associated with this event. This result is consistent with the results of Chen et al. [27].

### 3.4 Contingency information

**Figure 5:**
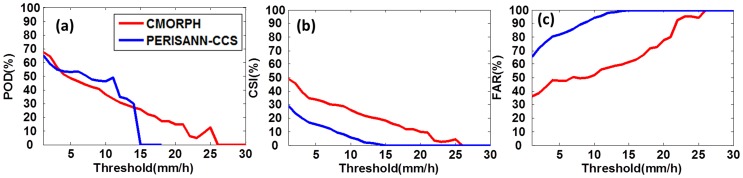
Contingency statistics for (a) POD, (b) CSI and (c) FAR. The rainfall threshold used is from 1 to 30 with interval of 1/h.

Figure 5 provides contingency information in terms of POD, CSI and FAR. Figure 5a shows PERSIANN-CCS has low POD and CSI for rainfall rates greater than 15 mm/h, while CMORPH has low POD and CSI when rainfall rates exceed 25 mm/h. This result provides further evidence that PERSIANN-CCS and CMORPH generally perform poorly in the detection of very high rainfall rates, and is consistent with information revealed by the PDFs shown in Figure 4.

## Summary and Conclusions

The primary objective of this study was to quantify the performance of the popular satellite-based QPE products CMORPH and PERSIANN-CCS for an extreme precipitation event over Beijing and surrounding regions, using a dense gauge network as ground truth. The satellite-based QPE products have been evaluated in terms of accumulated rainfall, time series rainfall, probability distribution and contingency metrics. Our results identified the following:

- Both CMORPH and PERSIANN-CCS were unable to capture the spatial rainfall pattern (CC<0.6, Figure 2) and misplaced the region of greatest precipitation intensity/accumulation.
- CMORPH captured the temporal variation of rainfall with high CC (0.92) over the large area HBT but could not resolve the temporal variation of rainfall over Beijing and the rainfall maximum (Figure 3a–c).
- PERSIANN-CCS underestimated the rainfall by more than 60% over the large area HBT, Beijing and rainfall center, and failed to resolve the temporal variation (Figure 2d–e, Figure 3a–c).
- PERSIANN-CCS was unable to detect high rainfall rate (>15 mm/h), and CMORPH had low probabilities of high rainfall rate (>25 mm/h) (Figure 4, 5).

This study suggests that satellite-based QPE products CMORPH and PERSIANN-CCS demonstrated poor performance in very heavy rainfall events, similar results can be found in studies of Dinku et al. (2010) [19], and Zhang (2012) as well as Chen et al. [27]. These two products tend to have limitations in terms of resolution and accuracy, especially for this type of extreme mid-latitude precipitation. Caution should be applied when CMORPH and PERSIANN-CCS are utilized for hydrological modeling and natural hazards (e.g. landslide) monitoring, because the data used to drive risk model cascades often form the dominant source of uncertainty within such model systems [33], [34]. However, we can envision that future Satellite-based QPE algorithms will be improved for hydrological and meteorological applications such as flood and landslide monitoring and forecasting, notably in the expected launch of Global Precipitation Measuring (GPM) mission in 2014 with dual-frequency radar onboard and better spatiotemporal coverage over the globe.
